# Validation of the Malay Version of the Affiliate Stigma Scale among Caregivers of Patients with Mental Illness

**DOI:** 10.21315/mjms2018.25.6.13

**Published:** 2018-12-28

**Authors:** Yap Siau Yun, Sharifah Zubaidiah Syed Jaapar, Nor Asyikin Fadzil, Kueh Yee Cheng

**Affiliations:** 1 Department of Psychiatry, School of Medical Sciences, Universiti Sains Malaysia, 16150 Kubang Kerian, Kelantan, Malaysia; 2 Hospital Universiti Sains Malaysia, Health Campus, USM, 16150 Kubang Kerian, Kelantan, Malaysia; 3 Unit of Biostatistics and Research Methodology, School of Medical Sciences, Universiti Sains Malaysia, 16150 Kubang Kerian, Kelantan, Malaysia

**Keywords:** factor analysis, caregivers of patients with mental illness, affiliate stigma, validity

## Abstract

**Background:**

Caregivers of patients with mental illness are exposed to stigma. The internalisation of this stigma among caregivers is known as affiliate stigma and can be measured by the Affiliate Stigma Scale (ASS). The aim of this study was to validate the Malay version of the ASS.

**Methods:**

A cross-sectional study was performed from May to December 2017 with 372 caregivers of patients with mental illness. The ASS was first translated into Malay using standard forward and backward translation procedures. The final version of the ASS-Malay (ASS-M) was completed by participants. The data analyses involved assessment of construct validity by exploratory factor analysis, confirmatory factor analysis and construct reliability.

**Results:**

The final model of the ASS-M consists of four factors with 21 items, as compared to the original version, which has three factors with 22 items. The results showed that the final model has good model fit based on RMSEA (0.065) and SRMR (0.055) and a satisfactory composite reliability (affective = 0.827, cognitive = 0.857, behaviour = 0.764, self-esteem = 0.861).

**Conclusion:**

The study showed that the four-factor, 21-item ASS-M model has good psychometric properties. The scale is valid and reliable for measuring affiliate stigma among caregivers of patients with mental illness in Malaysia.

## Introduction

Stigma is defined as a set of prejudicial attitudes, negative stereotypes, discrimination and biased social structures towards a certain group of people (1). The process of stigma starts with labelling and stereotyping, which in turn lead to separation, status loss and discrimination (2).

There are many ways of looking at stigma. Public stigma focuses on a community’s discrediting response to the stigmatised person, while holding a negative attitude or prejudice towards oneself is known as self-stigma (3–4). Courtesy stigma is the stigma experienced by family members or caregivers of a stigmatised person (5).

Affiliate stigma is the internalisation of negative stigma-related experiences by the family members of the stigmatised person (6). Affiliate stigma indirectly covers aspects of the caregiver’s self-stigma and their subsequent psychological responses of the associates. The result of this internalisation process affects the person’s cognition, affect, behaviour, self-esteem and self-efficacy.

The Affiliate Stigma Scale (ASS) consists of 22 items that measure the cognitive, affective and behavioural components of affiliate stigma (6). This scale was developed to study affiliate stigma among caregivers of people with intellectual disabilities or mental illness. The scale has been shown to have good psychometric properties, and its use has increased over the last few years. Furthermore, the ASS has already been validated and translated into different languages, including Chinese (6), Urdu (7), Hebrew (8), Hindi (9), Persian (10) and Amharic (11).

While stigma is commonly experienced by caregivers of patients with mental illness around the world, including Malaysia, a validated measurement scale is needed to assess affiliate stigma among caregivers in Malaysia. Thus, the aim of this study was to validate the Malay version of the ASS among caregivers of patients with mental illness in Kelantan, Malaysia.

## Materials and Methods

### Study Design and Procedures

This cross-sectional study was conducted in the psychiatric clinic at Universiti Sains Malaysia (USM) from May to December 2017. A total of 372 caregivers aged 18 and above consented to participate in the study. The estimated sample size for exploratory factor analysis (EFA) and confirmatory factor analysis (CFA) was calculated according to a standard size per domain set by Heir and colleagues (12). An estimated 20% non-response rate was also included for both EFA and CFA sample size determination. At the time of the study, the caregivers had been taking care of patients with mental illness (schizophrenia, mood disorder, anxiety disorder and intellectual disability) for at least 6 months. Participants who had major psychiatric illness were excluded from the study. The participants were recruited using non-probability convenience sampling. The study protocol was approved by the Human Research Ethics Committee of USM [USM/JEPeM/16120605].

### Measures

#### Affiliate Stigma Scale-Revised (ASS-R)

The ASS uses a 4-point Likert scale, ranging from 1 (strongly disagree) to 4 (strongly agree) (6), and includes 22 items assessing three domains (or subscales) of affiliate stigma: affective, cognitive and behavioural. The affective subscale consists of seven items (item 1, 4, 7, 10, 13, 16 and 19); the cognitive subscale also includes seven items (item 3, 6, 9, 12, 15, 18 and 21); and the behavioural subscale contains eight items (item 2, 5, 8, 11, 14, 17, 20 and 21). A higher mean score of the 22 items indicates a higher level of affiliate stigma. The ASS has good internal consistency [*α* = 0.94] for caregivers of mental illness and for exploratory factor analysis (6).

#### Instrument translation

**Figure 1:**
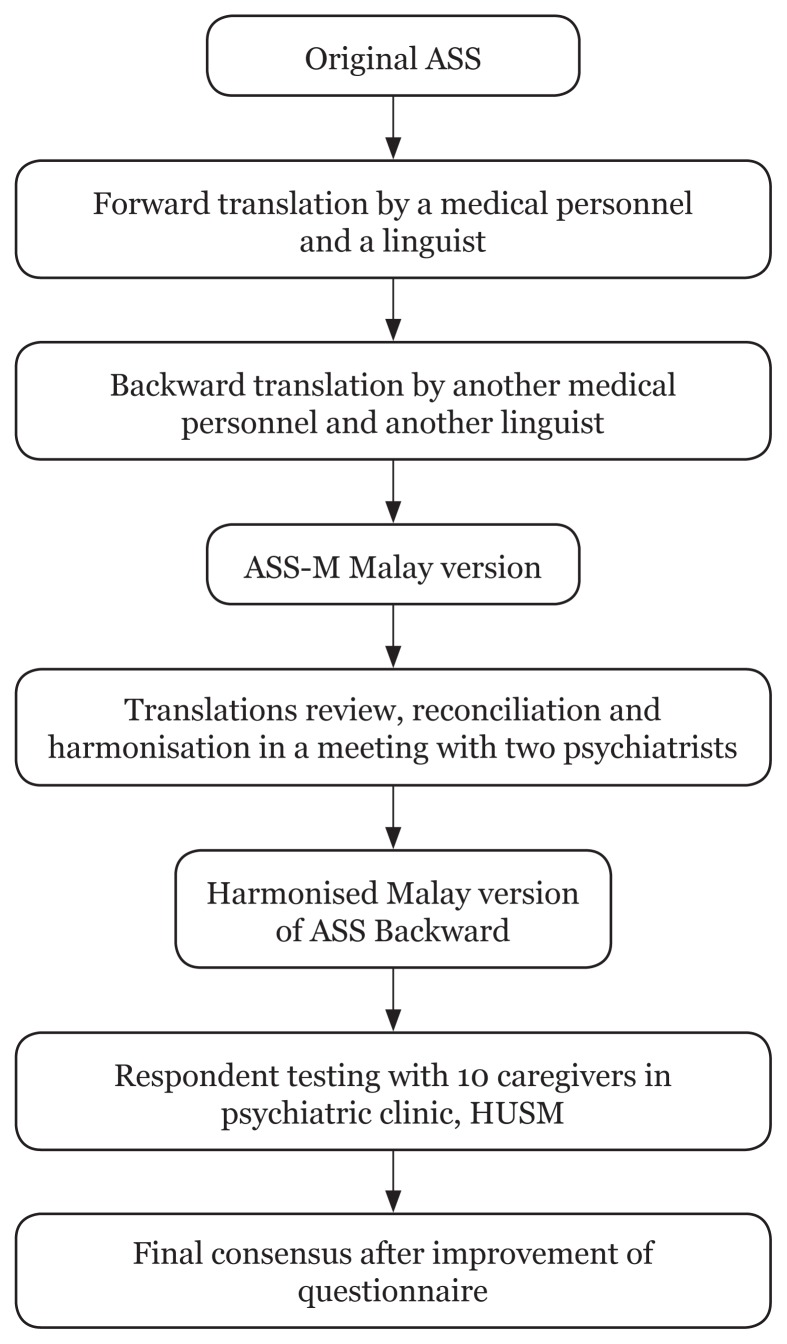
Translation process Note: ASS = Affiliate Stigma Scale; HUSM = Hospital Universiti Sains Malaysia

The original English language version of the ASS was translated into the Malay language using forward and backward translation by bilingual experts of Malay and English (see Figure 1). Two psychiatrists, who were competent bilingual speakers, reviewed both backward and forward translations, comparing each item in Malay to the corresponding item in the original English version. Expert panels assessed the contents of the questionnaire to be culturally appropriate to the Malaysian population. The final version in the Malay language, ASS-Malay (ASS-M), was pre-tested among 10 caregivers of patients with mental illness for clarity and comprehension. The participants were asked to answer the questions and to comment on the wording and presentation of the questionnaire. The results of the pre-test were found to be good, and therefore no modifications were necessary.

#### Statistical analysis

The Statistical Package for Social Sciences version 22.0 was used to analyse the data which included descriptive statistics of the respondents’ sociodemographic characteristics, EFA and internal consistency reliability. The acceptable cut-off value for the internal consistency reliability based on Cronbach’s alpha coefficient was ≥ 0.70 (12). A factor loading less than 0.3 was considered for removal of an item, and a factor with an Eigenvalue > 1.0 was accepted (12). The final model found in EFA was then confirmed by using CFA via Mplus 8 software (13).

The fitness of the model was assessed by the following indices: root mean square error of approximation (RMSEA) with an acceptable level of < 0.08; standardised root mean square residual (SRMR) with an acceptable level of < 0.08; Tucker-Lewis fit index (TLI) with an acceptable level of > 0.95; and finally, the comparative fit index (CFI) with an acceptable level of > 0.95 (12).

The construct reliability (CR) of the ASS-M was estimated by Raykov’s rho. A reliability based on Raykov’s rho of ≥ 0.70 was considered both reliable and acceptable (12). The acceptable cut-off value for the Cronbach’s alpha coefficient was also similar: ≥ 0.70 (12).

## Results

### Socio-demographic Characteristics of the Respondents

The participants (*n* = 132 for EFA; *n* = 240 for CFA) were mostly married (*n* = 86, 65.2%; *n* = 169, 70.4%) and female (*n* = 90, 68.2%; *n* = 160, 66.7%), and the mean age was nearly the same between EFA and CFA (43 years [SD = 15.3]; 44 years [SD = 16.2]). Most participants had received education up to the secondary level (*n* = 63, 48.5%; *n* = 130, 54.2%) but had a monthly income less than RM2,000 (*n* = 37, 28%; *n* = 87, 36%). The main caregivers participating in the study were parents of patients with mental illness (*n* = 52, 39.4%; *n* = 104, 43.3%) (Table 1).

### Exploratory Factor Analysis (EFA)

Principal axis factoring analysis with Promax rotation was conducted and resulted in a total of four factors with eigenvalues greater than one. The value of the Kaiser-Meyer-Olkin (KMO) measure of sampling adequacy test was excellent, at 0.92. Bartlett’s test of sphericity was significant, with *x*^2^ (231) = 2170.164, *P* < 0.01. All items loaded in a single dimension, with the value of factor loading higher than 0.30 (Table 2). Item 2 was deleted, as its factor loading was lower than 0.3.

All items were arranged based on the factor loading under the four factors extracted in this study (Table 3). Items with cross loading results were rearranged under the related factor after discussion with experts from the research team. Item 1, ‘I feel inferior ……’, had a factor loading that was slightly lower for the affective factor [0.328] than for the behaviour factor [0.418]. However, the research team decided to put item 1 under the affective factor, as this item was related to emotion more than to behaviour. Similar to item 1, both items 9 and 21 were placed under the cognitive factor despite their factor loadings [0.452; 0.487] being lower than those for self-esteem [0.456] and behaviour [0.564].

### Confirmatory Factor Analysis (CFA)

The four-factor model extracted from EFA was tested, and each item was allowed to load on its corresponding factor. The results of CFA are shown in Tables 4 and 5.

The initial model for the ASS-M had a good fit to the data based on fit indices of RMSEA and SRMR except for CFI and TLI (see Table 5). Further modification to the model was done to improve the fit indices.

The final ASS-M model displayed the following fit indices: RMSEA = 0.065, SRMR = 0.055, CFI = 0.904, TLI = 0.888. These results showed that two out of four fit indices (i.e., RMSEA and SRMR) were within the acceptable threshold despite modifications being made. No further modification was done because all the factor loadings (see Table 4) were above the recommended value and the items were found to be important, to remain in the constructs.

### Reliability

Composite reliability based on Raykov’s method indicated good internal consistency for the ASS-M: affective factor [*α* = 0.801], cognitive factor [*α* = 0.918], behaviour factor [*α* = 0.796] and self-esteem factor [*α* = 0.904] (Table 3). This demonstrates adequate evidence for the reliability of the Malay version of the ASS. The CR for the ASS-M was more than 0.7, as required (12).

## Discussion

In this study, exploratory and confirmatory factor analyses for the factor structure of the ASS-M were conducted. EFA was performed to extract the new factor structure from the dataset and compare it with the three-factor model, while CFA was conducted to assess the fitness of the new model.

The prominent sociodemographic characteristics of the participants in this study, i.e., female, middle-aged, married and parental caregivers, was a finding similar to that generated in studies on related subjects (14–15).

In comparison to the original ASS model, which has a three-factor construct, EFA in the present study produced a four-factor construct with the removal of one item: Q2 ‘I avoid communicating with a family member having mental illness/intellectual disability’. Q2 was considered inappropriate for the Malaysian population because relevant data have shown that most Malaysians prefer to talk than to keep silent when faced with a problem (16). Thus, it is likely that almost all caregivers would have responded to this item with ‘strongly disagree’, i.e., a score of 1.

The four factors differed from the original ASS with respect to item clustering. This could be due to the cultural adaptation of the original validated scale (17). Thus, the four factors are affective (item 1, 4, 13, 19), cognitive (item 3, 9, 11, 15, 16, 18, 21, 22), behaviour (item 5, 14, 17, 20) and self-esteem (item 6, 7, 8, 10, 12). The research team named the fourth factor ‘self-esteem’ based on the meanings of all items under it. This factor also correlates with many studies which have shown that self-stigma plays an important role in a stigmatised person’s self-esteem (18–19).

The standardised factor loadings yielded from EFA ranged between 0.32 and 0.86. These were higher than those in the original model’s factor loading, which ranged between 0.42 and 0.79 (6). The internal consistency of the ASS-M with a Cronbach’s alpha of 0.92 was comparable to the Cronbach’s alpha of 0.94 for the ASS (6). This was good, as its value was more than 0.7, as required (12).

Several fit indices were used to evaluate the goodness of fit of the model. For the final ASS-M model, the values of both RMSEA and SRMR were lower than 0.08 (12), indicating a good fit. The CFI and TLI indices did not reach the 0.95 cut-offs suggested by Hair et al. (12). However, Maiyaki (20) suggested that CFI values above 0.9 can indicate an acceptable fit. It has also been recommended that stringent criteria should be applied with caution (21).

All items achieved a satisfactory factor loading to their respective factors. Most items achieved a loading of more than 0.5—except for item 3, whose value [0.49] was slightly lower than 0.5. Based on these indices, this study had an acceptable fit to the four-factor model.

The four-factor ASS-M model proposed in this study has good psychometric properties and will be useful for researchers and health care providers in Malaysia to study the association and impact of affiliate stigma on caregivers of patients with mental illness, whose main spoken language is Malay. The ASS-M model will enable more interventions in the future to reduce the harmful effects of affiliate stigma.

We acknowledge that there were some limitations in the present study. The majority of participants in this study was Malays and using only one study site may hinder the generalisation of the results to other ethnicities in Malaysia. The data collection process, which relied on self-reported questionnaires, may have led to response bias. Additionally, no comparisons were made with other tools that also assess affiliate stigma among caregivers. Finally, the scale was administered only once in this study; hence, other important psychometric properties, such as test–retest reliability, could not be tested.

Future research should be expanded to caregivers of patients with mental illness in different hospitals in other Malaysian states to confirm the generalisability of the ASS-M instrument. A comparison with other tools, which would support the concurrent validity of the ASS-M, would generate increased value. An interviewer-rated tool would be more suitable as well, as it could reduce response bias. Test–retest reliability is recommended in future research, especially in prospective studies aimed at examining caregivers’ affiliate stigma levels at different times.

## Conclusion

This study showed that the four-factor, 21-item ASS-M model has good psychometric properties. The scale is valid and reliable for measuring affiliate stigma among caregivers of patients with mental illness in Malaysia.

## Figures and Tables

**Table 1:** Sociodemographic data of respondents for Exploratory Factor Analysis (*n* = 132) and Confirmatory Factor Analysis ( *n* = 240)

Variables	EFA		CFA	
	
	Frequency (%)	Mean (SD)	Frequency (%)	Mean (SD)
Age		43 (15.3)		44 (16.2)
Gender				
Male	42 (31.8)		80 (33.3)	
Female	90 (68.2)		160 (66.7)	
Race				
Malay	127 (96.2)		235 (97.9)	
Chinese	4 (3)		3 (1.3)	
Indian	1 (0.8)		-	
Others	-		2 (0.8)	
Marital Status				
Single	44 (33.3)		61 (25.4)	
Married	86 (65.2)		169 (70.4)	
Widow/Divorce	2 (1.5)		9 (3.8)	
Educational Status				
Primary	13 (9.8)		25 (10.4)	
Secondary	64 (48.5)		130 (54.2)	
Tertiary	53 (41.7)		85 (35.5)	
Occupation				
Student	8 (6.1)		5 (2.1)	
Government staff	30 (22.7)		54 (22.5)	
Private staff	24 (18.2)		25 (10.4)	
Housewife	32 (24.2)		64 (26.7)	
Self-employed	30 (22.7)		67 (27.9)	
Unemployed	8 (6.1)		25 (10.4)	
Monthly Income				
< 1,000	39 (29.5)		66 (27.5)	
1,000–1,999	37 (28)		87 (36.3)	
2,000–2,999	22 (16.7)		24 (10.0)	
3,000–3,999	10 (7.6)		20 (8.3)	
4,000–4,999	7 (5.3)		14 (5.8)	
> 5,000	17 (12.9)		29 (12.1)	
Median (IQR)	2200		2200	
Relationship with patient				
Parents	52 (39.4)		104 (43.3)	
Siblings	24 (18.2)		37 (15.4)	
Spouse	23 (17.4)		52 (21.7)	
Children	22 (16.7)		41 (17.1)	
Relatives	6 (4.5)		6 (2.5)	
Others	5 (3.8)		-	

**Table 2:** Item factor loading and communalities for Exploratory Factor Analysis (*n* = 132).

Item	Factor loading				Communalities

	1	2	3	4	
Q1	0.418			0.328	0.538
Q2	0.221				0.323
Q3		0.443	0.319		0.362
Q4				0.614	0.556
Q5	0.475				0.230
Q6			0.539		0.673
Q7			0.612		0.686
Q8			0.860		0.689
Q9		0.452	0.456		0.626
Q10			0.530		0.698
Q11		0.673	0.335		0.734
Q12			0.432	0.301	0.678
Q13				0.541	0.414
Q14	0.713			0.337	0.682
Q15		0.677		0.491	0.734
Q16	0.463	0.521			0.707
Q17	0.566				0.647
Q18		0.407			0.658
Q19		0.301		0.534	0.560
Q20	0.816				0.716
Q21	0.564	0.487			0.780
Q22		0.782			0.821

**Table 3:** Internal consistency reliability for Exploratory Factor Analysis (*n* = 132)

Factor	Item description	Cronbach’s alpha
Emotion		0.801
Q1	I feel inferior because I have a family member with mental illness/intellectual disability. *Saya berasa rendah diri kerana ada di kalangan keluarga saya yang menghidapi masalah penyakit mental/kurang upaya intelektual.*	
Q4	I feel emotionally disturbed because I have a family member with mental illness/intellectual disability. *Saya berasa terganggu dari segi emosi kerana mempunyai ahli keluarga yang menghidap penyakit mental / kurang upaya intelektual*	
Q13	I feel sad because I have a family member with mental illness/intellectual disability. *Saya berasa sedih kerana saya mempunyai ahli keluarga yang ada sakit mental/kurang upaya intelektual.*	
Q19	I feel that I am under great pressure because I have a family member with mental illness/intellectual disability. *Saya merasakan bahawa saya mengalami tekanan yang besar kerana saya mempunyai ahli keluarga yang menghidap penyakit mental / kurang upaya intelektual*	
Cognitive		0.918
Q3	Other people would discriminate against me if I am with a family member having mental illness/intellectual disability. *Orang lain akan mendiskriminasi saya kerana saya mempunyai ahli keluarga yang mempunyai masalah penyakit mental kurang upaya intelektual*	
Q9	People’s attitude towards me turns bad when I am together with a family member having mental illness/intellectual disability. *Sikap orang lain terhadap saya berubah kepada layanan yang buruk bila saya bersama-sama dengan ahli keluarga yang ada sakit mental/ kurang upaya intelektual*	
Q11	Given that I have a family member with mental illness/intellectual disability, I reduce contact with my friends and relatives *Memandangkan saya mempunyai ahli keluarga yang menghidap penyakit mental / kurang upaya intelektual, saya kurang berhubung dengan rakan dan saudara-mara saya*.	
Q15	Having a family member with mental illness/intellectual disability makes me think that I am incompetent compared to other people. *Mempunyai ahli keluarga yang menghidap penyakit mental / kurang upaya intelektual membuatkan saya berfikir bahawa saya tidak cekap berbanding dengan orang lain.*	
Q16	I worry that other people would know I have a family member with mental illness/ intellectual disability. *Saya bimbang sekiranya orang lain mengetahui bahawa saya mempunyai ahli keluarga yang ada sakit mental/ kurang upaya intelektual.*	
Q18	Having a family member with mental illness/intellectual disability makes me think that I am lesser to others *Mempunyai ahli keluarga yang menghidap sakit mental/ kurang upaya intelektual, membuatkan saya berfikir bahawa saya mempunyai kekurangan berbanding orang lain*	
Q21	Having a family member with mental illness/intellectual disability makes me lose face. *Mempunyai ahli keluarga yang menghidap sakit mental/ kurang upaya intelektual membuatkan saya berasa malu*	
Q22	Given that I have a family member with mental illness/intellectual disability, I reduce contact with the neighbours. *Memandangkan saya mempunyai ahli keluarga yang menghidap penyakit mental / kurang upaya intelektual, saya kurang berhubung dengan jiran-jiran.*	
Behaviour		0.796
Q5	I dare not tell others that I have a family member with mental illness/intellectual disability. *Saya tidak akan memberitahu kepada orang lain bahawa saya mempunyai ahli keluarga yang ada sakit mental/ kurang upaya intelektual*	
Q14	When I am with a family member having mental illness/intellectual disability, I would keep a relatively low profile. *Apabila saya bersama dengan ahli keluarga yang menghidap penyakit mental / kurang upaya intelektual, saya akan cuba tidak menojolkan diri*	
Q17	I reduce interacting with a family member with mental illness/intellectual disability. *Saya kurang berinteraksi bersama dengan ahli keluarga yang mempunyai sakit mental/ kurang upaya intelektual*	
Q20	I dare not to participate in activities related to mental illness/intellectual disability lest other people would suspect I have a family member with mental illness/intellectual disability. *Saya tidak rela melibatkan diri dengan aktiviti yang berkaitan sakit mental/ kurang upaya intelektual supaya dapat mengelakkan daripada disyaki mempunyai keluaraga yang menghidap sakit mental/ terencat akal.*	
Self-esteem		0.904
Q6	My reputation is damaged because I have a family member with mental illness/intellectual disability *Reputasi saya rosak kerana saya mempunyai ahli keluarga yang ada sakit mental/ kurang upaya intelektual.*	
Q7	The behavior of a family member with mental illness/intellectual disability makes me feel embarrassed *Kelakuan ahli keluarga yang menghidap penyakit mental /kurang upaya intelektual membuatkan saya berasa malu*	
Q8	I reduce going out with a family member with mental illness/intellectual disability. *Saya mengurangkan kekerapan saya keluar dengan ahli keluarga yang ada sakit mental/ kurang upaya intelektual*	
Q10	I feel helpless for having a family member with mental illness/intellectual disability. *Saya berasa tidak berdaya kerana mempunyai ahli keluarga yangada sakit mental/ kurang upaya intelektual.*	
Q12	Having a family member with mental illness/intellectual disability imposes a negative impact on me. *Mempunyai ahli keluarga yang menghidap penyakit mental / kurang upaya intelektual memberikan kesan negatif kepada saya*	

**Table 4:** Factor Loading of Confirmatory Factor Analysis for study model (*n* = 240)

Item	Standardised Factor Loading for Study Model		CR (95%CI)

	Initial	Final	
Affective			0.827 (0.779, 0.876)
Q1	0.688	0.684	
Q4	0.758	0.763	
Q13	0.728	0.729	
Q19	0.776	0.774	
Cognitive			0.857 (0.812, 0.902)
Q3	0.505	0.489	
Q9	0.665	0.660	
Q11	0.705	0.714	
Q15	0.777	0.768	
Q16	0.721	0.691	
Q18	0.727	0.713	
Q21	0.780	0.761	
Q22	0.777	0.779	
Behaviour			0.764 (0.693, 0.836)
Q5	0.493	0.481	
Q14	0.770	0.772	
Q17	0.762	0.764	
Q20	0.700	0.703	
Self-esteem			0.861 (0.818, 0.905)
Q6	0.762	0.758	
Q7	0.762	0.741	
Q8	0.687	0.683	
Q10	0.774	0.772	
Q12	0.821	0.829	

**Table 5:** Fit Indices for Affiliate Stigma Scale of study model

Models	RMSEA (90% CI)	SRMR	CFI	TLI
Study Model				
Initial	0.074 (0.064, 0.083)	0.058	0.876	0.857
Final	0.065 (0.056, 0.075)	0.055	0.904	0.888

Note: Correlation factor for Study Model: Q21 with Q16, Q9 with Q3; Q18 with Q15; Q7 with Q6. CFI = comparative fit index; TLI = Tucker–Lewis fit index; SRMR = standardised root mean square residual; RMSEA = root mean square error of approximation; CI = confidence interval
